# Socioeconomic Inequalities in Overweight and Obesity in Serbia: Data from 2013 National Health Survey

**DOI:** 10.3389/fphar.2017.00967

**Published:** 2018-01-08

**Authors:** Sekulic Marija, Vasiljevic Dragan, Radevic Svetlana, Djonovic Nela

**Affiliations:** ^1^Department of Hygiene and Ecology, Faculty of Medical Sciences, University of Kragujevac, Kragujevac, Serbia; ^2^Center of Hygiene and Human Ecology, Institute of Public Health Kragujevac, Kragujevac, Serbia; ^3^Department of Social Medicine, Faculty of Medical Sciences, University of Kragujevac, Kragujevac, Serbia

**Keywords:** socioeconomic inequalities, overweight, obesity, National Health Survey, Serbia

## Socioeconomic inequalities in overweight and obesity—impact on health overweight and obesity and their impact on health

Overweight and obesity represent the significant public health problem (Averett et al., 2008; Sánchez et al., 2017). Prevalence of the obesity in the world in 2014 reached 11% of men and 15% of women (NCD Risk Factor Collaboration, 2016), and it is assessed that 38% of the world adult population will be overweight up to 2030, and 20% will be obese (Hruby and Hu, 2015) In the USA more than of a third of the adult population is obese (35%), but more than of a two-thirds of population is overweight (69%) (NCD Risk Factor Collaboration, 2016). By the evaluation of the obesity prevalence in European countries, showing variations from one country to another, the higher prevalence of obesity in the Central, Eastern and South Europe was noticed. The prevalence of obesity in most cases was less in European countries than in the United States of America (Berghöfer et al., 2008; Flegal et al., 2010). In our country the increase of the obese persons is also recorded, the figure from the research of the Serbian population health in 2013 showed the presence of obesity in 21.9% of persons older than 20 years (Ministry of Health of the Republic of Serbia, 2014).

Overweight and obesity were correlated with the occurrence of numerous chronic diseases, contributing to an increase in total morbidity and mortality, as well as to the serious economic pressure of a family and the increase in costs within a society worldwide (Mc Donald et al., 2015; Wang et al., 2016). Obesity represents a risk factor for the occurrence of numerous chronic non-contagious diseases, such as cardiovascular, diabetes mellitus of type 2, carcinomas; it results in the increase in the mortality rate all over the world (National Institutes of Health, 1998; Whitlock et al., 2009; World Health Organization, 2009; Stanković and Jašović-Gaši, 2010; Wormser et al., 2011).

Numerous factors contribute to the occurence of the obesity such as the following: the old age, gender, nationality, socio-economic level, marital status (El Rhazi et al., 2010; Pampel et al., 2012). Socio-economic differences are obvious in nutrition, giving thus an explanation for the presence of social inequalities in health (Alkerwi et al., 2012). People with the high social-economic status have a higher probability for the healthier habits in nutrition in relation to the people with the worse socio-economic status, who are not able to follow complete nutritive recommendations and guidelines in nutrition, resulting in their worse health state (James et al., 1997). Therefore, the main concern of the public health should be both social inequality and diet quality in order to acquire the healthy dietary behaviors (Alkerwi et al., 2015).

In Serbia, like in many other countries in transition, the existing socio-economic inequalities in health have not been studied enough and have not received full attention in the policy of the public health. Undeveloped countries from the Balkans have to face numerous health challenges as well. The leading causes of morbidity and mortality, absenteeism, disability, and premature death are chronic non-communicable diseases (mainly cardiovascular diseases, cancers, chronic respiratory diseases, and diabetes) that are mostly preventable if adequate measures are implemented (Jakovljevic and Varjacic, 2017). Health status of the population was mostly affected by population aging and negative socio-economic developments during the last decade (Jakovljević, 2017). All countries of the Western Balkans region are facing growing difficulties in the provision of sustainable health care financing and equitable access to medical care for their citizens (Jakovljevic, 2013). Transitional reform processes of national health systems across Eastern European and Balkan societies have been present for almost two and half decades (Jakovljevic et al., 2017). Despite enormous invested efforts and resources many of the key features of past, such as heavy hospital-based system of medical service provision and presence of large state controlled health insurance funds have remained present in most of these countries (Jakovljevic et al., 2011; Jakovljevic and Souliotis, 2016).

## The data report methods

### Public data set description—Serbian 2013 national health survey

Data used are from the Third National Survey conducted by the Ministry of Health of the Republic of Serbia in 2013. The survey was conducted in accordance with a type of the cross-sectional study on the territory of the Republic of Serbia and it did not include the population living on the territory of the Autonomous Region of Kosovo and Metohija. In the third research, a methodology applied was the methodology and instruments of the European Health Research—the second wave (EHIS-wave 2) (Eurostat, 2013).

From 10,089 households in total, 6,500 of them gave their consent for the participation in the research, so that the rate of household response was 64.4%. From 16,474 registered members of households older than 19, over 14,623 of them agreed to be interviewed, with the response rate of 88.9%. From the total number of people who gave the consent for the polling, 13,922 accepted to fill the questionnaire (the response rate of 94.1%). The data used in this particular study was the data on the adult older than 19.

The data set has been submitted in a public repository Figshare and it is available on: https://figshare.com/s/ef49f5fe703c247252ad Data has been uploaded as Excel file while questionnaires are in PDF formats. Readers are free to access and reuse these data at the links provided above.

### Description of national survey outcomes

The study included 13,922 persons aged 19 and over, more women (54.0%), than men (46.0%). The average age of persons was 51.41 ± 17.7 years. The largest number of persons were from an urban environment (56.4%), were married (64.7%), had secondary school level of education (54.5%), were employed (32.6%) and they belonged to the poorest classes of the population (22.5%).

In the studied sample the BMI ranged from 12.5 to 55.9 kg/m^2^, with the average value of BMI of 26.6 kg/m^2^. The largest percentage of persons (37.2%) were in a category of normal weight persons, then followed by the overweight (36.9%), and the obese (23.6%). The least number of persons were in a category of the underweight (2.3%). The percentage of overweight and obese (BMI ≥ 25) persons (60.5%) was 1.5 times higher in relation to the percentage of the normal weight persons. Obesity of I degree (BMI = 30.0–34.9 kg/m^2^) was present in 17.1% of persons, obesity of II degree (BMI = 35.0–39.9 kg/m^2^) at 5.0% and obesity of III degree (BMI ≥40 kg/m^2^) had 1.5% of persons.

By the observation in accordance with demographic characteristics, there is a statistically significant difference in the level of the weight status. The analysis by the gender showed that women were in the higher percentage with normal weight in relation to the men (40.7%: 33.3%). Overweight was the more frequent in men (43.5%: 31.2), the obesity was in a larger percentage present in women (24.9%: 22.1) (χ^2^ = 259.513; *p* ≤ 0.001). The average value of BMI is statistically significantly higher in men (26.77) in relation to women (26.36) (*t* = 4.289; *p* ≤ 0.001). Prevalence of the number of persons who were (overweight and obese) was gradually increased with the persons' years of life and the highest was in the age of 65–74 years (74.5%), after those years it was decreased (χ^2^ = 1322.316; *p* ≤ 0.001).

The highest prevalence of the overweight (39.2%) and the obese (30.6%) was among widowers and widows, while the lowest prevalence of obesity (8.4%) was among persons who were never married or who were in the common-law marriages (χ^2^ = 878.822; *p* ≤ 0.001).

Person who lived in an urban environment (22.1%) had significantly lower prevalence of the obesity in the relation to the persons living in other settlements (25.6%) (χ^*p*^ = 27.346; *p* ≤ 0.001). The largest percentage of the overweight and obese persons with BMI (≥25) was present in South and Eastern Serbia (62.2%) and Vojvodina (61.8%) (χ^2^ = 25.325; *p* = 0.003).

Prevalence of the overweight and obese persons with BMI (≥25) is in an inverse proportion to the education level, even 2/3 of persons with the lowest level of education are overweight and obese with BMI (≥25), 67.9% while that percentage at persons with the higher level of education is significantly lower 54.2%. As regards persons with the lowest level of education, the prevalence of the obesity (31.7%) was almost two times higher when comparing to persons with the higher level of education (17.1%). Employed persons had lower prevalence of the obesity (18.9%) in relation to the economic inactive persons (28.7%) and the unemployed persons (20.7%) (χ^2^ = 119.611; *p* ≤ 0.001). There is a statistically significant difference in the obesity prevalence in relation to the material status of persons, persons who belong to the category of the poorest have a significantly higher prevalence of the obesity (26.4%) in relation to persons who belong to the wealthiest class of population (18.4%) (χ^2^ = 93.278; *p* ≤ 0.001; Table 1).

**Table 1:** Distribution weight status by demographic and socioeconomic characteristics.

**Variables**	**Weight status (BMI)**				***p***
	**Underweight**	**Normal weight**	**Overweight**	**Obesity**	
**GENDER**					
Men	1.2	33.3	43.5	22.1	<0.001
Women	3.3	40.7	31.2	24.9	
**AGE (YEARS)**					
20–34	5.7	58.3	26.9	9.1	<0.001
35–44	1.8	41.9	36.7	19.7	
45–54	1	33.2	39.2	26.7	
55–64	0.9	25.5	41.0	32.6	
65–74	1.2	24.2	41,0	33.5	
75+	2.5	33.5	40.2	23.8	
**REGION**					
Vojvodina	2.1	36.1	37.7	24.1	0.003
Belgrade	2.4	40.9	35.3	21.4	
Sumadia and Eastern Serbia	2.4	36.9	36.9	23.8	
South and East Serbia	2.3	35.6	37.4	24.8	
**TYPE OF SETTLEMENT**					
Urban	2.2	38.6	37.1	22.1	<0.001
Other (rural)	2.4	35.5	36.5	25.6	
**MARITAL STATUS**					
Unmarried	6.0	58.6	26.9	8.4	<0.001
Married	1.3	33.5	39.0	26.2	
Widowed	2.7	27.6	39.2	30.6	
Divorced	2.3	42.2	34.8	20.8	
**EDUCATIONAL LEVEL**					
Primary	2.4	29.7	36.2	31.7	<0.001
Secondary	2.4	39.1	37.1	21.4	
Higher	2.0	43.9	37.1	17.1	
**EMPLOYMENT STATUS**					
Employed	1.4	41.7	37.9	18.9	<0.001
Unemployed	3.6	42.0	33.8	20.7	
Inactive	2.3	31.3	37.7	28.7	
**HOUSEHOOLD WEALTH**					
I (the poorest)	2.9	35.2	35.6	26.4	<0.001
II poor	2.7	34.7	36.7	26.0	
III middle	1.8	35.9	38.2	24.0	
IV wealthy	1.7	39.1	37.3	21.9	
V (the wealthiest)	2.3	42.6	36.7	18.4	

At the multi-variant model, adjusted to all observed demographic and socio-economic variables as the most important factors, correlated with the overweight and obese, the gender, age, education, and marital status of persons were isolated.

Female persons has 1.7 times a higher risk for the overweight and obese in relation to men (OR = 1.705). By increasing age the number of the overweight and obese persons becomes higher, and the risk is the highest in the age group 55–64 (OR = 3.26) and 65–75 years (OR = 3.23), even threetimes higher in relation to the youngest age group. Proportion of the persons with BMI (≥25) is in an inverse proportion to the level of education. Persons with the lower education were 1.5 times more frequently overweight and obese (OR = 1.48) in relation to those with the higher education level. When the marital status is in question, persons who were never married/in common-law marriage had by 48% less chance to be overweight and obese (OR = 0.52) in relation to persons who were in a marriage/common-law marriage (Table 2).

**Table 2:** Odds ratios (OR) and 95% confidence intervals (CI) for overweight and obesity depending on demographics and socioeconomic characteristics.

	**Univariate model**	***p***	**Multivariate model**	***p***
	**OR (95%CI)**		**OR(95%CI)**	
**GENDER**				
Women	1		1	
Men	1.429 (1.331–1.535)	<0.001	1.705 (1.574–1.846)	<0.001
**AGE GROUPS**				
19–34	1		1	
35–44	2.180 (1.942–2.448)	<0.001	1.726 (1.521–1.958)	<0.001
45–54	3.212 (2.857–3.611)	<0.001	2.461 (2.161–2.802)	<0.001
55–64	4.671 (4.164–5.239)	<0.001	3.260 (2.838–3.745)	<0.001
65–74	4.982 (4.370–5.680)	<0.001	3.229 (2.709–3.849)	<0.001
75+	3.099 (2.708–3.547)	<0.001	1.959 (1.626–2.360)	<0.001
**MARITAL STATUS**				
Married/common–law marriage	1		1	
Unmarried/common law marriage	0.310 (0.280–0.342)	<0.001	0.523 (0.463–0.590)	<0.001
Divorce, separation, death of a partner	1.075 (0.976–1.183)	0.142	1.001 (0.898–1.115)	0.985
**TYPE OF SETTLEMENT**				
Other (rural)	1		1	
Urban	0.875 (0.815–0.940)	<0.001	0.981 (0.896–1.073)	0.669
**EDUCATIONAL LEVEL**				
Higher	1		1	
Secondary	1.211(1.100–1.334)	<0.001	1.231 (1.106–1.371)	<0.001
Primary	1.847 (1.655–2.061)	<0.001	1.486 (1.300–1.699)	<0.001
**HOUSEHOLD**				
Wealthy class	1		1	
Poor class	1.273(1.177–1.378)	<0.001	0.960 (0.863–1.067)	0.447
Middle class	1.236 (1.120–1.363)	<0.001	1.078 (0.968–1.201)	0.173
**EMPLOYMENT STATUS**				
Inactive	1		1	
Employed	0.643 (0.593–0.698)	<0.001	0.907 (0.803–1.025)	0.118
Unemployed	0.612 (0.558–0.670)	<0.001	0.894 (0.792–1.009)	0.068

### Comparison with published evidence

According to the data of the World Health Organization, 39% of the adult of the aged 18 and more were overweight in 2016, while 13% of them were obese^1^. Prevalence of the obesity in Serbia (23.6%) is almost two times higher than the prevalence in the world (12%), the prevalence of the overweight (36.9%) is on the similar level (39%). In comparison with the prevalence in the European region (23% of obese, 36% of overweight) the difference is not so pronounced as in relation to the World level. According to the results of the Global Status Report on non-communicable diseases from 2014, the only region in the World that has more prevalence of the overweight and obesity than Europe is the region of America (61% of overweight or obese, and 27% of obese persons). The prevalence is the lowest in South-Eastern Asia region where 5% of the population is obese, and 17% are overweight. Globally observed, countries with the highest prevalence of the overweight (BMI ≥ 25) are the Cook Islands (80.0%), Palau (78.4%), (Nauru 77.0%), (Qatar 76.6%), and the Marshall Islands (74.9%). On the other side are Timor–Leste (12.1%), Burundi (13.4), Afghanistan (13.9%), Nepal (16.4%) and Ethiopia (16.5) (World Health Organization 2014). In most European countries in the neighborhood of Serbia, the prevalence of the overweight and obese is similar: Slovenia (64.8%), Austria (56.6%), Greece (64.9%), Bulgaria (63.6%), Croatia (62.9%), Hungary (63.3%), Montenegro (58.4%), Romania (60.8%), The former Yugoslav Republic of Macedonia (57.5%), Bosnia and Herzegovina (54.6%) and Albania (53.5%). The lowest percentage of the obesity in 2014 among the population aged 18 and more were recorded in Romania (9.4%) and Italy (10.7%), Holland (13.3%), Belgium, and Sweden (14.0%). On the opposite end of a scale, the highest percentage of the obese was on Malta (26.0%), Latvia (21.3%), Hungary (21.2%), Estonia (20.4%), and the Great Britain (20.1%)^2^. All those variations among countries, in the prevalence of the overweight and obese can be explained by the socio-demographic and cultural differences, as well as differences in the methodological approach used during data collecting.

In 2014, 11% of men and 15% of women all around the world were obese. In European, Eastern Mediterranean Region and regions of America, over 50% of women were overweight or obese, and in all three regions around a half of overweight women are obese (25% European region, 24% in the region of Eastern Mediterranean, 30% in the region of America). In all regions of the World Health Organization, women are more obese than men (World Health Organization, 2014). The situation in Serbia is similar to most European countries, the prevalence of overweight is more frequent at men, the prevalence of the obesity is more frequent at women (Kuntz and Lampert, 2010; World Health Organization, 2014; Ogden et al., 2015). Without regard to the fact that in most countries the obesity is more frequent at women, in some European countries (Croatia, Denmark, Ireland, Italy, Spain, Switzerland) the obesity is more frequent in men (Mascie-Taylor and Goto, 2007). In Serbia during 2014, the overweight and obese (BMI ≥ 25) in male persons, aged 18 was even more represented (65.5%), while in female persons that percentage was 56.1%. The findings are similar to the values of surrounding European countries, the percentage of adult persons (older than 18 and more) the overweight varied in 2014 among (36.1%) in Italy and (55.2%) in Malta for women and among (53.6%) in Holland and (67.5%) in Croatia for men^3^. In the population aged 18 and more, during 2014, the least percentage of women who were considered as obese was recorded in Romania (9.7%), Italy (10.3%), Cypress (12.9%), and Austria (13.4%), the least percentage of the obese men was in Romania (9.1%), Italy (11.3%), Holland (11.6%), and Sweden (13.6%). The percentage of the obese women during 2014 in Serbia was 24.9%, and this points out that the percentage is similar as in some European countries, Malta (23.9%), Latvia (23.3%), Estonia (21.5%), and the Great Britain (20.4%), among men the presence of obesity (21.1%) in Serbia is similar to the findings in Malta (28.1%), Hungary (22.0%), Slovenia (21.0%), and Croatia (20.7%) in the same period^3^.

Years of life and the level of education were significantly correlated with the overweight and obesity, irrespective of the gender. Prevalence of the overweight person number was gradually increased with the persons' years of life (Andreyeva et al., 2007; Ministry of Health of the Republic of Serbia, 2014). It has ascending course up to 60, then the weight starts to decrease (Grujić et al., 2017), documented by the findings of the multinational research of Europeans and population research in USA (Villareal et al., 2005; Grujić et al., 2017). A fall in the obesity after 60 years can be explained by the decrease of motion, loss of an appetite in older people, as well as the fact that with aging the muscular mass decreases and it becomes replaced with the adipose tissue (Grujić et al., 2017).

In accordance with the marital status data show that the lowest prevalence of obesity (8.4%) was among persons who were never in a marriage or a common-law marriage, in comparison with other categories (the married, widowers, and divorced). The study in Greece (Tzotzas et al., 2010) and in the National Health and Nutrition Examination Survey (NHANES) in America (Sobal et al., 2009) showed the similar values. As opposed to data in Serbia where the highest percentage of the obese is present in the category of widowers, with the explanation that the status of a widower leads to the occurrence of stress, and it may have as a consequence the obesity (Umberson et al., 2009), the study conducted in Turkey documented the highest representation of the obese in persons involved in marriage as opposed to other categories (Kilicarslan et al., 2006). Comparing statuses of the married and unmarried, the collected data indicates that the prevalence of the obese is significantly higher in the married persons, also supported by numerous studies in the Western (Erem et al., 2004; Tur et al., 2005) and Eastern society (Janghorbani et al., 2008). The reason for that may be probably the fact that married couples have more regular meals, consume the food of the high energetic density, that they are not worried whether they will be attractive to a partner if they are overweight, as well as that they are discouraged to exercise due to the numerous family duties (Sobal et al., 2003). As opposed to those findings that document that there is a significant correlation between the obesity and marital status, one Dutch study did not find a significant correlation between the given points (van Lenthe et al., 2000). On the other side, a Swiss study points out that the life in marriage acts protective against the increase of the body mass and that a possible explanation for it can lie in the fact that the life in marriage has positive effects on behavior, in relation to the health and promotion of the healthy life styles (Guerra et al., 2015).

The low level of education in Serbia was correlated with the higher risk for the overweight and obesity (BMI ≥ 25), which means that the prevalence of obesity is increased with the decrease of the education level. It might be that the lower education level increases a psychosocial distress, having as a result the excessive food consumption, reduced physical activity and the occurrence of the obesity (Bennett et al., 2008). That an invert correlation between level of education and obesity exists the data of EPIC Panacea study indicate, including data for an adult population from 10 European countries (Hermann et al., 2011) and studies of Cohen et al. (2013). Therefore, the high level of education has the positive impact on our health, leads to increase of interests for knowledge on health, offers higher ability of information usage in relation to the health, offers obvious perception of the risk supported by specific styles of life and in that way it inclines to the healthy form of behavior (Yoon et al., 2006; Devaux et al., 2011).

Men and women with lower socio-economic status (SES) are often obese in relation to the category of population with the higher SES. In comparison of the income, data proves that persons of the average material status were less affected by the obesity in relation to the groups of the lower status, while they were much affected by the obesity in relation to the group with the higher material status, approved by the findings Benjamin Kuntz et al. (Kuntz and Lampert, 2010). One of the possible explanations on an impact of the socio-economic factors on the obesity is that above mentioned factors have impact on an inequality for accessibility to the healthy food. The poor categories of the population are in less opportunity to afford the nutritive food quality. They can mostly afford caloric food, rich in fats and sugars, poor in nutritive ingredients, and as a consequence it can have the increased rate of obesity, while the population with the higher incomes can afford nutritive food quality (Cohen et al., 2013; Rao et al., 2013).

## Conclusive remarks

Socio-economic inequalities in the health represent an important challenge for the health policy, because not only do they represent social inequality, but by solving health problems of the poorest groups of population one can have an impact on improving the health state of the population in entirety. Obesity can be prevented through multi-sector population interventions that proclaim physical activity and consumption of the nutritive important food, during entire life.

## Author contributions

All authors listed, have made substantial, direct, and intellectual contribution to the work, and approved it for publication.

### Conflict of interest statement

The authors declare that the research was conducted in the absence of any commercial or financial relationships that could be construed as a potential conflict of interest.

## Footnotes

^1^World Health Organisation. *Obesity and Overweight*. World Health Organisation Available online at: http://www.who.int/mediacentre/factsheets/fs311/en/ (cited October 25, 2017).

^2^Eurostat. *European Health Interview Survey*. Available online at: http://ec.europa.eu/eurostat/documents/2995521/7700898/3-20102016-BP-EN.pdf/c26b037b-d5f3-4c05-89c1-00bf0b98d646

^3^Statistical Office of the European Union. *Overweight and Obesity – BMI Statistics. Statistics Explained*. Available online at: http://ec.europa.eu/eurostat/statistics-explained/index.php/Overweight_and_obesity_-_BMI_statistics
